# Associations of the Endothelial Activation and Stress Index with breast cancer prevalence and mortality based on NHANES 2001 to 2018

**DOI:** 10.1097/MD.0000000000047777

**Published:** 2026-02-28

**Authors:** Shaoqun Huang, Ming Gao, Hongyang Gong

**Affiliations:** a Department of Oncology Surgery, Fuzhou Traditional Chinese Medicine Hospital, Fuzhou City, Fujian Province, China; b Department of Gastroenterology, The First Hospital of Jilin University, Changchun, Jilin, China; c Department of Physiology, College of Medicine, Chosun University, Gwangju, Republic of Korea.

**Keywords:** breast cancer, Endothelial Activation and Stress Index, endothelial malfunction, mortality, NHANES

## Abstract

This study investigated the association between the Endothelial Activation and Stress Index (EASIX) and both the prevalence and mortality of breast cancer (BC). Further, it explored the potential mediating role of the Systemic Inflammation Response Index (SIRI). Data were obtained from the National Health and Nutrition Examination Survey 2001 to 2018. Weighted logistic regression models were used to examine the association between EASIX and BC prevalence, while mediation analysis evaluated the contribution of SIRI. Weighted Cox proportional hazards models estimated hazard ratios (HRs) and 95% confidence intervals (CIs) for BC mortality, and Kaplan–Meier curves compared survival across EASIX levels. Restricted cubic spline regression assessed linearity, and subgroup analyses tested consistency across covariates. Among 21,329 participants, 593 BC cases were identified. Each 1-unit increase in EASIX was associated with a 39% higher BC risk (odds ratios [ORs] = 1.39, 95% CI: 1.07–1.82, *P* = .016). Compared with the lowest tertile (T1), the risk was elevated in T2 (OR = 1.61, 95% CI: 1.15–2.26, *P* = .006) and markedly higher in T3 (OR = 3.07, 95% CI: 2.28–4.14, *P* < .001). SIRI showed a modest but significant mediating effect, explaining 2.26% of the association (*P* = .034). No significant interactions were detected. In Cox models, each 1-standard deviation increase in EASIX was associated with a 56% higher risk of all-cause mortality (HR = 1.56, 95% CI: 1.47–1.65, *P* < .001) and a 104% higher risk among BC patients (HR = 2.04, 95% CI: 1.40–2.98, *P* < .001). Compared with T1, all-cause mortality increased by 59% in T2 (HR = 1.59) and more than 3-fold in T3 (HR = 3.11). BC-specific mortality was also higher in T3 (HR = 1.16, 95% CI: 1.05–1.27, *P* = .012). Restricted cubic spline analyses confirmed a linear positive association between EASIX and both BC prevalence and mortality. Elevated EASIX was significantly associated with higher BC prevalence and mortality, partly mediated by systemic inflammation. EASIX may serve as a valuable biomarker for risk assessment and prognosis in BC.

## 1. Introduction

Breast cancer (BC) is the most frequently diagnosed malignancy among women worldwide and remains one of the leading causes of cancer-related mortality in this population.^[1]^ Epidemiological data indicate that approximately 2.3 million new cases of BC were reported globally in 2020, accounting for nearly one-quarter of all newly diagnosed cancers in women.^[2]^ The incidence of BC is projected to increase by 40% by 2040.^[3]^ The development of BC is multifactorial, with established risk factors including genetic predisposition, hormonal imbalance, and unfavorable lifestyle behaviors.^[4]^ Modifiable factors such as obesity, physical inactivity, alcohol consumption, and smoking have also been strongly linked to elevated BC risk.^[5]^ Despite advances in surgical techniques, targeted therapy, and immunotherapy that have improved survival in certain patients, substantial challenges remain in early detection, precision screening, and individualized prognostic assessment of BC.^[6]^ Emerging evidence suggests that chronic inflammation and endothelial dysfunction play pivotal roles in the initiation and progression of BC.^[7]^ Chronic inflammation promotes malignant transformation by regulating tumor-associated signaling pathways, whereas endothelial dysfunction disrupts vascular homeostasis, contributing to tumor angiogenesis, invasion, and metastasis.^[8]^ This study was designed as a cross-sectional analysis to comprehensively investigate the association between BC and endothelial function, to evaluate the potential value of endothelial status in BC risk stratification and prognostic prediction.

Molecular interactions between tumor cells and endothelial cells play a critical role in tumor angiogenesis.^[9]^ In BC, dysregulation of angiogenic mechanisms drives uncontrolled tumor growth and progression.^[10]^ The Endothelial Activation and Stress Index (EASIX), a novel composite biomarker derived from lactate dehydrogenase (LDH), creatinine, and platelet counts, provides a convenient and cost-effective tool for assessing endothelial dysfunction and microvascular injury.^[11]^ Initially introduced for prognostic prediction in allogeneic hematopoietic stem cell transplantation,^[11]^ EASIX has since been extended to risk stratification and outcome assessment in multiple diseases. Previous studies have demonstrated its prognostic utility in acute pancreatitis,^[12]^ acute kidney injury among critically ill cancer patients,^[13]^ coronary artery disease,^[14]^ and acute myocardial infarction.^[15]^ Moreover, EASIX has been validated in infectious and inflammatory conditions^[16]^ as well as in hematologic and solid malignancies, including multiple myeloma, small-cell lung cancer, and diffuse large B-cell lymphoma.^[16–18]^ Despite these findings, research on the prognostic relevance of EASIX in BC remains limited. Therefore, this study sought to utilize data from the National Health and Nutrition Examination Survey (NHANES) to examine, for the 1st time, the association between EASIX, BC prevalence, and all-cause mortality in BC patients.

Chronic systemic inflammation has been shown to contribute to tumor initiation and progression by releasing cytokines, altering immune cell distribution, and activating aberrant signaling pathways.^[19]^ The Systemic Inflammation Response Index (SIRI), calculated from neutrophil, monocyte, and lymphocyte counts, integrates inflammatory and immune responses into a single metric.^[20]^ In recent years, SIRI has been identified as a significant prognostic marker across multiple cancers, including pancreatic, gastric, hepatocellular, and thyroid cancers, where elevated levels were associated with poor survival outcomes.^[21]^ SIRI has also been suggested as a valuable indicator of therapeutic response in BC patients receiving neoadjuvant treatment; however, its role in BC development and progression remains unclear.^[22]^ Accordingly, the present study further aimed to evaluate the potential mediating role of SIRI in the relationship between EASIX and BC, thereby providing novel evidence on the interplay between systemic inflammation, endothelial dysfunction, and BC risk.

## 2. Materials and methods

### 2.1. Study population and design

Data for this study were obtained from the NHANES, conducted by the National Center for Health Statistics (NCHS), Centers for Disease Control and Prevention. NHANES is designed to comprehensively assess the health and nutritional status of the non-institutionalized U.S. population.^[23]^ Since 1999, NHANES has employed a complex, multistage, stratified probability sampling method, with data collected in 2-year cycles to ensure national representativeness.^[24]^ Information was obtained through household interviews, physical examinations, and laboratory tests, encompassing demographic characteristics, dietary intake, health conditions, and biochemical indicators. The survey protocol was approved by the NCHS Research Ethics Review Board, and written informed consent was obtained from all participants or their proxies.^[25]^ Publicly available datasets can be accessed at the Centers for Disease Control and Prevention website (https://www.cdc.gov/nchs/nhanes).

**Figure 1.**
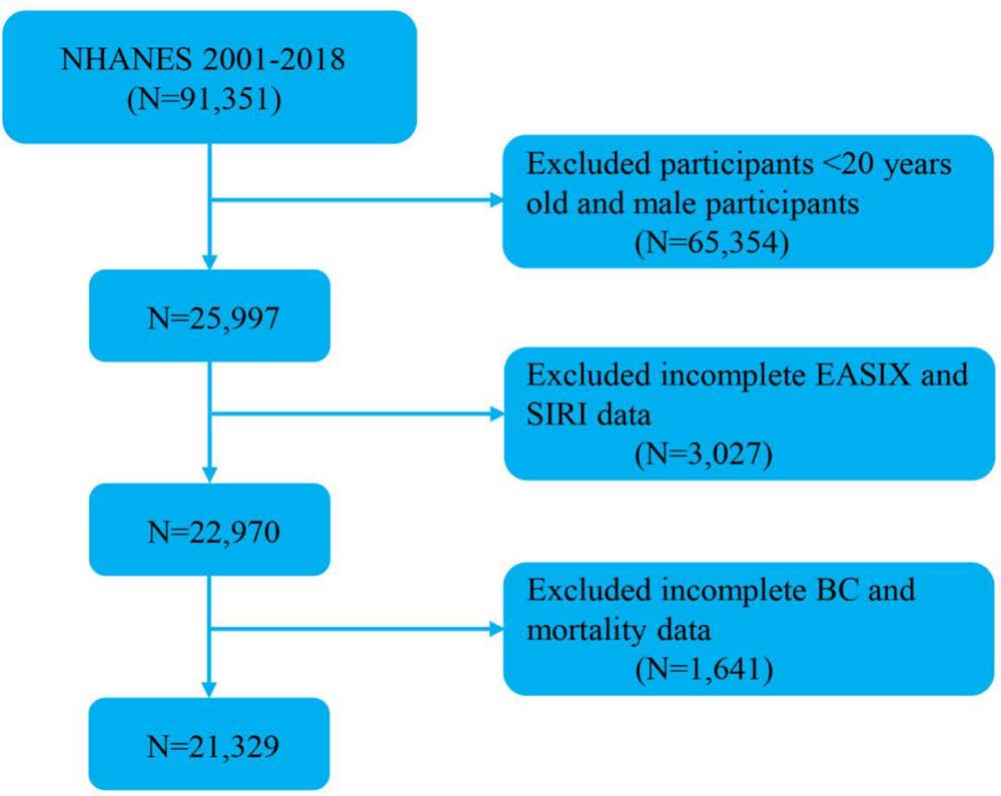
A flow diagram of eligible participant selection in the National Health and Nutrition Examination Survey. BC = breast cancer; EASIX = Endothelial Activation and Stress Index; SIRI = Systemic Inflammation Response Index.

This study included 9 consecutive NHANES cycles (2001–2018). Of 91,351 participants initially enrolled, males and individuals younger than 20 years or with restricted data access were excluded (N = 65,354). Participants lacking sufficient data for the calculation of EASIX or SIRI (N = 3027) were also excluded, as were those missing covariate information or mortality data (N = 1641). The final analytic cohort comprised 21,329 cancer survivors (Fig. 1).

### 2.2. Assessment of BC

BC status was determined using the NHANES Medical Conditions Questionnaire.^[26]^ Participants were asked: “Have you ever been told by a doctor or other health professional that you have cancer or any malignancy?” Respondents answering “yes” were further asked to specify the cancer type. Participants self-reporting BC only, with no history of other malignancies, were classified as the BC group, while those reporting “no” were classified as the non-BC group. Individuals reporting multiple cancers or responding “refused” or “don’t know” to cancer type were excluded.

### 2.3. Measurement of EASIX

The EASIX was calculated as follows: $EASIX=\frac{LDH\left(U/L\right)\times Creatinine\left(mg/dL\right)}{Platelet\;count\left(10^{9}/L\right)}$.^[27]^ Blood samples were collected in Mobile Examination Centers (MECs) following standardized protocols and analyzed in central laboratories under strict quality control. LDH was measured by monitoring the conversion of pyruvate to L-lactate, creatinine by the Jaffe rate method, and platelet counts by automated hematology analyzers.^[28]^

### 2.4. Definition of SIRI

SIRI was calculated using complete blood count data obtained from the Beckman–Coulter Automated Hematology Analyzer DxH 900 (Beckman–Coulter, Brea). Cell counts were reported in 10^3^/µL. SIRI was defined as: $SIRI=\frac{Neutrophil\;count\times Monocyte\;count}{Lymphocyte\;count}$.^[29]^

### 2.5. Covariates

Potential confounders were selected based on prior literature and clinical relevance.^[30–32]^ Demographic covariates included age, race/ethnicity (Hispanic, non-Hispanic Black, non-Hispanic White, and others), education level (<high school, ≥high school), marital status, and poverty income ratio (PIR, categorized as <1.3 or ≥1.3 according to NHANES guidelines). Clinical covariates included histories of diabetes, hypertension, and hyperlipidemia. Details of covariate data collection are provided in Table S1, Supplemental Digital Content, https://links.lww.com/MD/R418.

### 2.6. Mortality outcomes collection

Mortality outcomes were determined using the NCHS Public-Use Linked Mortality Files, which link NHANES participants to the National Death Index (https://ftp.cdc.gov/pub/Health_Statistics/NCHS/datalinkage/linked_mortality/). Follow-up time was calculated from the NHANES examination date until the date of death or December 31, 2019, whichever occurred first. The primary outcome was all-cause mortality.

### 2.7. Statistical analysis

Given the use of hematological variables, MEC sample weights were applied to account for the complex survey design and non-response bias.^[33]^ For 2001 to 2018 data, weights were calculated as 1/9 × WTMEC2YR.^[34]^ Continuous variables were expressed as weighted means with standard errors and compared using *t* tests. Categorical variables were expressed as weighted percentages and compared using chi-square tests. Baseline characteristics were summarized by BC status.

Multivariable logistic regression models were employed to estimate odds ratios (ORs) and 95% confidence intervals (CIs) for the association of EASIX and SIRI (as continuous variables or tertiles) with BC prevalence. Three models were analyzed: Model 1, unadjusted; Model 2, adjusted for age, education, marital status, PIR, and race/ethnicity; and Model 3, further adjusted for diabetes, hypertension, and hyperlipidemia. Tests for trend were conducted by treating EASIX and SIRI tertiles as ordinal variables. Restricted cubic spline (RCS) analyses were performed to assess potential nonlinear relationships. Subgroup analyses and interaction tests with key covariates were conducted, with results visualized using forest plots. Mediation analysis was applied to evaluate the direct effect of EASIX on BC risk and the indirect effect mediated by SIRI.

For survival analyses, multivariable Cox proportional hazards models were used to examine associations of EASIX (continuous and tertiles) with BC-specific and all-cause mortality, estimating hazard ratios (HRs) and 95% CIs. Model specifications were the same as above. Kaplan–Meier survival curves were plotted across EASIX tertiles, and RCS analysis was used to explore nonlinear relationships with all-cause mortality. All analyses were performed using R software (version 4.3.1). A 2-sided *P*-value < .05 was considered statistically significant.

## 3. Results

### 3.1. Population characteristics

A total of 21,329 participants were included in the final analysis, representing more than 979 million individuals in the U.S. population. Among them, 593 participants reported a history of BC, corresponding to an estimated 2.88 million BC cases nationwide. Detailed descriptive statistics stratified by BC status are presented in Table 1. Demographic characteristics indicated that 68% of BC cases were aged ≥60 years and 84% were non-Hispanic White. Compared with non-BC participants, those with BC were more likely to be widowed, divorced, or separated, and exhibited higher prevalences of hypertension, diabetes, and hyperlipidemia (all *P* < .05). In addition, levels of both EASIX and SIRI were significantly elevated in the BC group (*P* < .001).

**Table 1:** Baseline characteristics of all participants were stratified by BC, weighted.

Characteristic	Overall, N = 97,916,725 (100%)	Non-BC, N = 95,028,332 (97.1%)	BC, N = 2888,393 (2.9%)	*P*-value
No. of participants in the sample	21,329	20,736	593	–
Age (%)				<.001
20–40	39,156,675 (40%)	39,053,914 (41%)	102,761 (3.6%)	
41–60	36,117,587 (37%)	35,302,847 (37%)	814,741 (28%)	
>60	22,642,463 (23%)	20,671,572 (22%)	1,970,891 (68.4%)	
Race (%)				<.001
Non-Hispanic White	65,214,958 (67%)	62,774,895 (67%)	2,440,062 (84%)	
Non-Hispanic Black	11,784,904 (12%)	11,599,153 (12%)	185,751 (6.4%)	
Other	12,873,833 (13%)	12,699,314 (13%)	174,519 (6.0%)	
Mexican American	8,043,031 (8%)	7,954,970 (8%)	88,061 (3.6%)	
Married/live with partner (%)				.039
No	38,449,776 (39%)	37,158,150 (39%)	1,291,626 (45%)	
Yes	59,428,198 (61%)	57,831,430 (61%)	1,596,768 (55%)	
Education level (%)				.332
Below high school	15,909,348 (16%)	15,483,939 (16%)	425,408 (15%)	
High School or above	81,934,500 (84%)	79,471,515 (84%)	2,462,985 (85%)	
PIR (%)				<.001
Poor	21,341,634 (23%)	20,953,108 (24%)	388,526 (15%)	
Not poor	69,964,521 (77%)	67,731,722 (76%)	2,232,799 (85%)	
Hypertension (%)				<.001
No	63,845,629 (65%)	62,662,243 (66%)	1,183,386 (41%)	
Yes	34,065,277 (35%)	32,360,269 (34%)	1,705,007 (59%)	
Diabetes (%)				<.001
No	85,998,507 (88%)	83,790,563 (88%)	2,207,944 (76%)	
Yes	11,918,219 (12%)	11,237,769 (12%)	680,449 (24%)	
Hyperlipidemia (%)				<.001
No	28,885,984 (30%)	28,346,542 (30%)	539,442 (19%)	
Yes	69,030,742 (70%)	66,681,790 (70%)	2,348,951 (81%)	
EASIX (mean (SE))	0.42 (0.31)	0.42 (0.31)	0.55 (0.31)	<.001
EASIX, tertile (%)				<.001
T1	32,639,891 (33%)	32,218,861 (34%)	421,030 (14%)	
T2	32,635,425 (34%)	31,863,904 (33%)	771,521 (27%)	
T3	32,641,409 (33%)	30,945,567 (33%)	1,695,842 (59%)	
SIRI (mean (SE))	1.20 (0.82)	1.19 (0.82)	1.43 (0.97)	<.001
SIRI, tertile (%)				<.001
T1	32,660,492 (34%)	32,005,545 (34%)	654,948 (23%)	
T2	32,567,089 (33%)	31,698,792 (33%)	868,296 (30%)	
T3	32,689,145 (33%)	31,323,995 (33%)	1,365,150 (47%)	

Mean (SE) for continuous variables: the *P*-value was calculated by the weighted Students *T* test.

Percentages (weighted N, %) for categorical variables: the *P*-value was calculated by the weighted chi-square test.

BC = breast cancer; EASIX = Endothelial Activation and Stress Index; PIR = Poverty Income Ratio; SIRI = Systemic Inflammation Response Index.

### 3.2. Association of EASIX and SIRI levels with the odds of BC

Multivariable logistic regression analyses were conducted to examine the associations of EASIX and SIRI with BC risk (Table 2). In the fully adjusted model (Model 3), EASIX was positively associated with BC risk; each 1-unit increase in EASIX corresponded to a 39% higher risk of BC (OR = 1.39, 95% CI: 1.07–1.82, *P* = .016). When categorized into tertiles, participants in T2 had a 61% higher risk (OR = 1.61, 95% CI: 1.15–2.26, *P* = .006), and those in T3 had a more than 3-fold risk (OR = 3.07, 95% CI: 2.28–4.14, *P* < .001) compared with T1. A significant dose–response trend across EASIX tertiles was observed in all models (*P* for trend < .001).

**Table 2:** Association between EASIX, SIRI, and BC.

Characteristics	Model 1 [OR (95% CI)]	*P*-value	Model 2 [OR (95% CI)]	*P*-value	Model 3 [OR (95% CI)]	*P*-value
EASIX–BC						
Continuous	1.66 (1.21–2.28)	.002	1.65 (1.17–2.33)	.005	1.39 (1.07–1.82)	.016
Tertile						
T1	1 (ref.)		1 (ref.)		1 (ref.)	
T2	1.85 (1.34–2.56)	<.001	1.64 (1.17–2.29)	.004	1.61 (1.15–2.26)	.006
T3	4.19 (3.22–5.46)	<.001	3.69 (2.79–4.88)	<.001	3.07 (2.28–4.14)	<.001
* P* for trend	<.001		<.001		<.001	
SIRI–BC						
Continuous	1.28 (1.19–1.37)	<.001	1.21 (1.12–1.32)	<.001	1.16 (1.05–1.27)	.002
Tertile						
T1	1 (ref.)		1 (ref.)		1 (ref.)	
T2	1.34 (1.00–1.79)	.048	1.32 (0.97–1.78)	.076	1.24 (0.92–1.68)	.150
T3	2.13 (1.61–2.82)	<.001	1.84 (1.35–2.49)	<.001	1.61 (1.19–2.18)	.002
* P* for trend	<.001		<.001		.002	

Model 1: no covariates were adjusted.

Model 2: age, education level, marital status, PIR, and race were adjusted.

Model 3: age, education level, marital status, PIR, race, hypertension, diabetes, and hyperlipidemia were adjusted.

BC = breast cancer; CI = confidence interval; EASIX = Endothelial Activation and Stress Index; OR = odds ratio; PIR = poverty income ratio; SIRI = Systemic Inflammation Response Index.

Similarly, SIRI was positively associated with BC risk after adjustment for confounders. Each 1-unit increase in SIRI was associated with a 16% higher risk (OR = 1.16, 95% CI: 1.05–1.27, *P* = .002). In tertile analyses, participants in T3 had a 61% increased risk compared with T1 (OR = 1.61, 95% CI: 1.19–2.18, *P* = .002), with a significant upward trend across tertiles (*P* for trend < .05). In addition, a significant positive correlation between EASIX and SIRI was observed in Model 3 (*P* = .019; Table 3).

**Table 3:** Multivariate linear regression of EASIX and SIRI.

Characteristics	β	95% CI	*P*-value
EASIX–SIRI	0.05	(0.01–0.10)	.019

Adjusted for age, education level, marital status, PIR, race, hypertension, diabetes, and hyperlipidemia.

CI = confidence interval; EASIX = Endothelial Activation and Stress Index; OR = odds ratio; PIR = poverty income ratio; SIRI = Systemic Inflammation Response Index.

**Figure 2.**
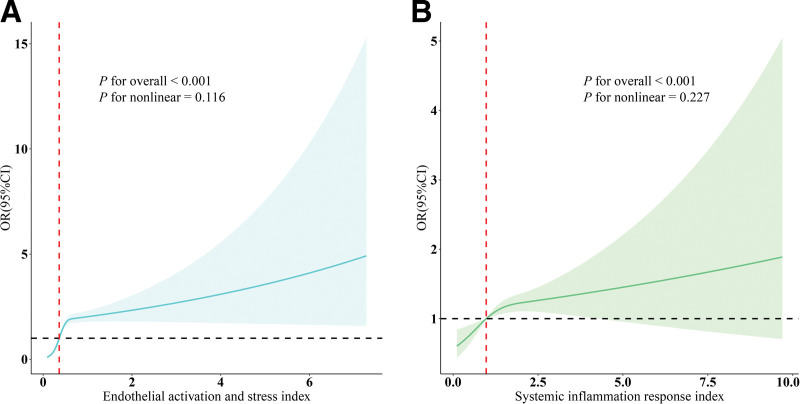
Dose–response relationships between EASIX, SIRI, and BC. (A) EASIX–BC; (B) SIRI–BC. OR (solid lines) and 95% confidence levels (shaded areas) were adjusted for age, education level, marital status, PIR, race, hypertension, diabetes, and hyperlipidemia. BC = breast cancer; EASIX = Endothelial Activation and Stress Index; ORs = odds ratios; PIR = poverty income ratio; SIRI = Systemic Inflammation Response Index.

RCS analyses based on fully adjusted models further demonstrated linear dose–response relationships of EASIX (*P* for overall < .001; *P* for nonlinear = .116; Fig. 2A) and SIRI (*P* for overall < .001; *P* for nonlinear = .227; Fig. 2B) with BC risk, indicating that higher EASIX and SIRI levels were linearly associated with increased BC prevalence.

### 3.3. Subgroup analysis

**Figure 3.**
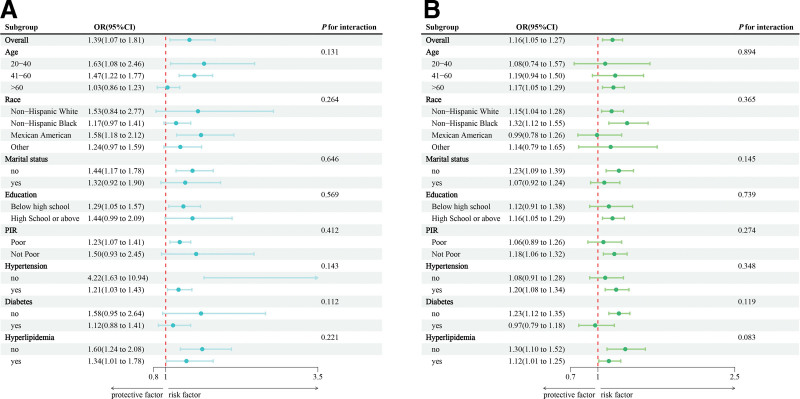
Subgroup analysis between EASIX, SIRI, and BC. (A) EASIX–BC; (B) SIRI–BC. ORs were calculated per 1-unit increase in EASIX, and each standard deviation increased in SIRI. Analyses were adjusted for age, education level, marital status, PIR, race, hypertension, diabetes, and hyperlipidemia. BC = breast cancer; EASIX = Endothelial Activation and Stress Index; ORs = odds ratios; PIR = poverty income ratio; SIRI = Systemic Inflammation Response Index.

Subgroup analysis was conducted stratified by age, education, marital status, PIR, race, hypertension, diabetes, and hyperlipidemia (Fig. 3). Significant positive associations were observed between EASIX and BC risk among participants aged 40 to 60 years, Mexican Americans, unmarried individuals, those with lower education, higher poverty, no history of hypertension, and no hyperlipidemia (all *P* < .05). Stronger associations between SIRI and BC risk were identified among participants aged ≥ 60 years, non-Hispanic Whites, unmarried individuals, and those without diabetes or hyperlipidemia (all *P* < .05). No significant interactions between EASIX or SIRI and the stratification variables were detected.

### 3.4. Mediation analysis

**Figure 4.**
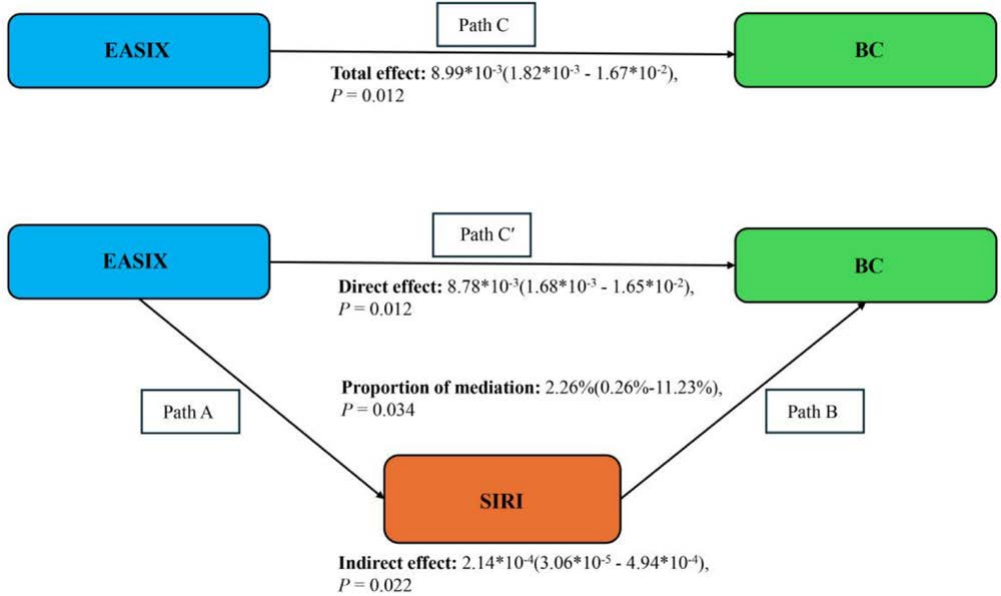
Schematic diagram of the mediation effect analysis. Path C indicates the total effect; path C′ indicates the direct effect. The indirect effect is estimated as the multiplication of paths A and B (path A*B). The mediated proportion is calculated as indirect effect/(indirect effect + direct effect) × 100%. Analyses were adjusted for age, education level, marital status, PIR, race, hypertension, diabetes, and hyperlipidemia. BC = breast cancer; EASIX = Endothelial Activation and Stress Index; PIR = poverty income ratio; SIRI = Systemic Inflammation Response Index.

Mediation analysis was performed to explore whether systemic inflammation mediated the association between EASIX and BC risk. As shown in Figure 4, after adjustment for all confounders, SIRI partially mediated the association, accounting for 2.26% of the total effect (*P* = .034).

### 3.5. Association between EASIX and all-cause mortality

Cox proportional hazards models were applied to evaluate the association of EASIX with all-cause mortality (Table 4). After adjustment for confounders, higher EASIX was consistently associated with elevated mortality risk, whether modeled as a continuous variable or by tertiles (*P* for trend < .05). In Model 3, each 1-unit increase in EASIX was associated with a 56% higher risk of all-cause mortality in the overall cohort (HR = 1.56, 95% CI: 1.47–1.65, *P* < .001), and a 104% higher risk among BC survivors (HR = 2.04, 95% CI: 1.40–2.98, *P* < .001). In tertile analyses, participants in T2 had a 59% higher mortality risk compared with T1 (HR = 1.59, 95% CI: 1.31–1.92, *P* < .001), and those in T3 had a more than 3-fold higher risk (HR = 3.11, 95% CI: 2.68–3.62, *P* < .001). Among BC survivors, those in T3 also had a significantly increased risk of all-cause mortality (HR = 1.16, 95% CI: 1.05–1.27, *P* = .012).

**Table 4:** HRs (95% CIs) are used for all-cause mortality, according to the EASIX.

Characteristics	Model 1 [HR (95% CI)]	*P*-value	Model 2 [HR (95% CI)]	*P*-value	Model 3 [HR (95% CI)]	*P*-value
All participants						
Continuous	1.62 (1.50–1.75)	<.001	1.64 (1.53–1.75)	<.001	1.56 (1.47–1.65)	<.001
Tertile						
T1	1 (ref.)				1 (ref.)	
T2	1.71 (1.43–2.06)	<.001	1.73 (1.43–2.09)	<.001	1.59 (1.31–1.92)	<.001
T3	4.58 (3.96–5.31)	<.001	4.34 (3.72–5.06)	<.001	3.11 (2.68–3.62)	<.001
* P* for trend	<.001		<.001		<.001	
BC						
Continuous	2.10 (1.47–2.99)	<.001	2.33 (1.63–3.33)	<.001	2.04 (1.40–2.98)	<.001
Tertile						
T1	1 (ref.)				1 (ref.)	
T2	0.81 (0.45–1.46)	.483	0.83 (0.41–1.68)	.602	0.90 (0.48–1.68)	.589
T3	1.34 (1.17–1.52)	<.001	1.10 (1.03–1.18)	<.001	1.16 (1.05–1.27)	.012
* P* for trend	<.001			<.001	.018	

Model 1: No covariates were adjusted.

Model 2: age, education level, marital status, PIR, and race were adjusted.

Model 3: age, education level, marital status, PIR, race, hypertension, diabetes, and hyperlipidemia were adjusted.

BC = breast cancer; CI = confidence interval; EASIX = Endothelial Activation and Stress Index; HR = hazard ratio; PIR = poverty income ratio.

RCS analysis revealed a significant association between EASIX and mortality risk, though no evidence of nonlinearity was detected (*P* for overall = .011; *P* for nonlinear = .947; Figure S1, Supplemental Digital Content, https://links.lww.com/MD/R418). Kaplan–Meier survival curves confirmed these findings, showing markedly lower survival among participants in the highest EASIX tertile compared with those in the lowest tertile (Figure S2, Supplemental Digital Content, https://links.lww.com/MD/R418). Collectively, elevated EASIX was consistently associated with higher all-cause mortality among BC survivors.

## 4. Discussion

Using nationally representative data from NHANES (2001–2018), this study included 21,329 participants, reflecting over 979 million U.S. residents. To our knowledge, this is the 1st investigation to comprehensively examine the association between the EASIX and both BC prevalence and mortality. The findings demonstrated significant positive linear relationships of EASIX with BC risk and all-cause mortality. Notably, these associations remained robust after full adjustment for potential confounders. Moreover, systemic inflammatory biomarkers partially mediated these associations. Collectively, the results suggest that EASIX may serve as a practical and reliable biomarker for identifying individuals at higher risk of BC and for predicting prognosis among BC survivors.

Tumor angiogenesis is a hallmark of cancer, providing nutrients and oxygen to malignant cells and facilitating tumor progression and metastasis.^[35]^ Unlike the orderly, tightly connected endothelial monolayers of normal vasculature, tumor vessels are typically disorganized, immature, and highly permeable, with disrupted tight and adherens junctions that undermine vascular homeostasis.^[36,37]^ The observed positive association between elevated EASIX and BC risk may be attributable to endothelial damage, which disrupts the balance between pro-angiogenic and anti-angiogenic factors.^[38]^ In addition, the hypoxic tumor microenvironment stimulates the release of vascular endothelial growth factor, angiopoietins, and other signaling molecules, which not only promote pathological angiogenesis but also directly activate and injure endothelial cells.^[39]^ Endothelial damage exposes collagen and tissue factor, triggering coagulation pathways, consuming platelets, and thereby fostering a vicious cycle that promotes tumor progression.^[40]^

Our mediation analysis further indicated that systemic inflammation partially accounts for the association between EASIX and BC. Chronic low-grade inflammation is a well-established feature of BC, with the tumor microenvironment enriched in immune cells, pro-inflammatory cytokines, and reactive oxygen species.^[41]^ Pro-inflammatory mediators such as TNF-α and IL-6 can activate endothelial cells, leading to the upregulation of adhesion molecules, including intercellular adhesion molecule-1, vascular cell adhesion molecule-1, P-selectin, and E-selectin.^[42]^ These molecules not only facilitate leukocyte migration and infiltration into tumor tissue^[43]^ but also provide docking sites for tumor cell adhesion and extravasation, thereby accelerating distant metastasis. Furthermore, activated endothelial cells themselves secrete additional inflammatory mediators, amplifying a self-sustaining loop that perpetuates NF-κB activation and other key signaling pathways. This process promotes epithelial–mesenchymal transition, enhances tumor invasiveness, and facilitates metastatic dissemination.^[44,45]^

The EASIX score, as a composite biomarker, integrates 3 readily available routine parameters: LDH, serum creatinine, and platelet count. Elevated LDH reflects the enhanced glycolytic metabolism and proliferative activity of tumor cells driven by the Warburg effect, while also indicating enzymatic signals released from tissue necrosis and disruption of cell membrane integrity, collectively representing tumor burden and tissue damage.^[46]^ Increased serum creatinine (or decreased eGFR) not only serves as an indicator of renal function but also reflects cancer-related systemic microvascular injury and endothelial dysfunction,^[47]^ suggesting a systemic environment unfavorable for tissue perfusion, which may exacerbate hypoxia and promote tumor progression. Platelets, on the one hand, facilitate immune evasion and metastatic colonization of circulating tumor cells by encapsulation^[48]^; on the other hand, as carriers of multiple angiogenic regulators (e.g., vascular endothelial growth factor, PDGF), they directly support tumor growth by mediating angiogenesis and maintaining vascular integrity.^[49,50]^ Thus, EASIX integrates endothelial activation, inflammation, and coagulation, providing an efficient and cost-effective tool to evaluate endothelial injury, tumor aggressiveness, and prognosis in BC patients.

This study is the first to demonstrate a significant positive association between the EASIX and both BC prevalence and all-cause mortality, and to identify a modifying role of SIRI in this relationship. Several strengths should be highlighted. First, the data were derived from a large, nationally representative cohort, and the use of NHANES sampling weights allows the findings to be generalized to the U.S. BC population. Second, extensive adjustment for demographic, clinical, and laboratory covariates strengthened the validity of effect estimates. Third, consistent associations were observed across stratified subgroup analyses, underscoring the robustness of EASIX as a prognostic biomarker for BC. Finally, the application of multiple analytical strategies, including restricted cubic spline modeling, revealed important linear relationships that may have been overlooked by traditional linear approaches.

Nonetheless, several limitations should be acknowledged. First, the cross-sectional design precludes causal inference between EASIX and mortality risk. Second, although we adjusted for a range of demographic, socioeconomic, and cardiometabolic covariates (including age, education level, marital status, PIR, race/ethnicity, hypertension, diabetes, and hyperlipidemia) residual confounding cannot be completely excluded. Some established BC-specific risk factors, such as detailed reproductive history, menopausal status, hormone therapy use, and family history of BC, were not available or were inconsistently collected across NHANES survey cycles and therefore could not be included in the multivariable models. In addition, information on certain lifestyle factors, including body mass index and smoking status, was not incorporated into the primary analyses, which may have introduced unmeasured confounding. Future studies with more comprehensive clinical and reproductive data are warranted to further validate our findings and clarify the independent association between the EASI and BC outcomes. Importantly, NHANES does not collect detailed cancer-specific prognostic information, such as tumor stage at diagnosis, treatment modalities, or recurrence status. Consequently, these key determinants of BC survival could not be incorporated into the mortality models. The absence of these variables may limit the interpretability of the observed associations between the EASI and mortality among BC survivors, and the results should therefore be interpreted with caution. Future studies with more comprehensive clinical oncology data are warranted to further clarify these relationships. Third, EASIX was derived from a single time-point measurement and therefore does not capture longitudinal changes or their potential prognostic implications. Future prospective studies with repeated measurements are warranted to validate its predictive value over time. Moreover, BC assessment in NHANES relied on self-reported questionnaires rather than standardized clinical diagnostic criteria, which may have introduced misclassification bias. Fourth, as all participants were from the United States, external validation in populations of diverse geographic and ethnic backgrounds, including Asian cohorts, is essential to establish the broader applicability of EASIX. Finally, NHANES provides only all-cause mortality data, without cause-specific mortality (e.g., cardiovascular or infection-related deaths). Further investigations incorporating cause-specific mortality are required to clarify differential associations between EASIX and distinct clinical endpoints. Additionally, although the mediation analysis identified a statistically significant indirect effect, the proportion mediated (2.26%) was small, suggesting a modest effect size with limited biological and clinical relevance. This finding should therefore be interpreted cautiously and viewed as hypothesis-generating rather than indicative of a dominant or clinically actionable mechanistic pathway.

## 5. Conclusion

Based on NHANES data, this study is the 1st to demonstrate a positive association between EASIX and both BC prevalence and mortality. Higher EASIX levels were significantly associated with increased risks of BC prevalence and death, with inflammatory markers partially mediating these associations. EASIX may serve as a practical biomarker for prognostic assessment in BC. However, further validation across diverse populations and regions is required to confirm its generalizability.

## Acknowledgments

We sincerely appreciate the NHANES database for all the data.

## Author contributions

**Conceptualization:** Shaoqun Huang, Ming Gao, Hongyang Gong.

**Data curation:** Shaoqun Huang, Ming Gao, Hongyang Gong.

**Formal analysis:** Ming Gao, Hongyang Gong.

**Funding acquisition:** Shaoqun Huang.

**Investigation:** Shaoqun Huang, Hongyang Gong.

**Methodology:** Ming Gao, Hongyang Gong.

**Resources:** Hongyang Gong.

**Software:** Hongyang Gong.

**Supervision:** Shaoqun Huang.

**Validation:** Shaoqun Huang.

**Writing – review & editing:** Shaoqun Huang.

**Writing – original draft:** Hongyang Gong.

## Supplementary Material


