# Supplementary material for: Associations of the Endothelial Activation and Stress Index with breast cancer prevalence and mortality based on NHANES 2001 to 2018
Source: Medicine (Baltimore). 2026 Feb 28;105(9):e47777. doi: 10.1097/MD.0000000000047777 (PMC12956168; doi:10.1097/MD.0000000000047777)
Supplement: Supplementary file 1 [file medi-105-e47777-s001.docx]

**Supplementary Material**

**Table S1.** Definition of variables involved in this study.

| Variables | Description in NHANES |
| --- | --- |
| Age | Divided into three groups: 20-40 years old, 41-60  years old, >60 years old |
| Race | Mexican American, Non-Hispanic Black, Non-Hispanic White, Other Race |
| Educational level | Below high school, High School or above |
| Marital status | Yes: Married/Living with partner |
| PIR | Poor: <1.3; Not Poor:>=1.3 |
| Hypertension | The diagnostic criteria consist of self-reported hypertension history, the utilization of antihypertensive medication, a systolic blood pressure (SBP) ≥ 140mmHg, or a diastolic blood pressure (DBP) ≥ 90mmHg |
| Diabetes | Diabetes was defined as a history of previous diabetes, HbA1c level ≥6.5%, or fasting blood glucose level ≥126 mg/dL |
| Hyperlipidemia | (1) Triglyceride (TG) levels ≥150 mg/dl (1.7 mmol/L);(2) Total cholesterol (TC) levels ≥200 mg/dl (5.18 mmol/L);(3) Low-density lipoprotein (LDL) levels ≥130 mg/dl (3.37 mmol/L);(4) High-density lipoprotein (HDL) levels: Men: <40 mg/dl (1.04 mmol/L); Women: <50 mg/dl (1.30 mmol/L) ;(5) Individuals taking cholesterol-lowering drugs are also considered hyperlipidemia. |

PIR, poverty income ratio.

**Supplementary Figures**


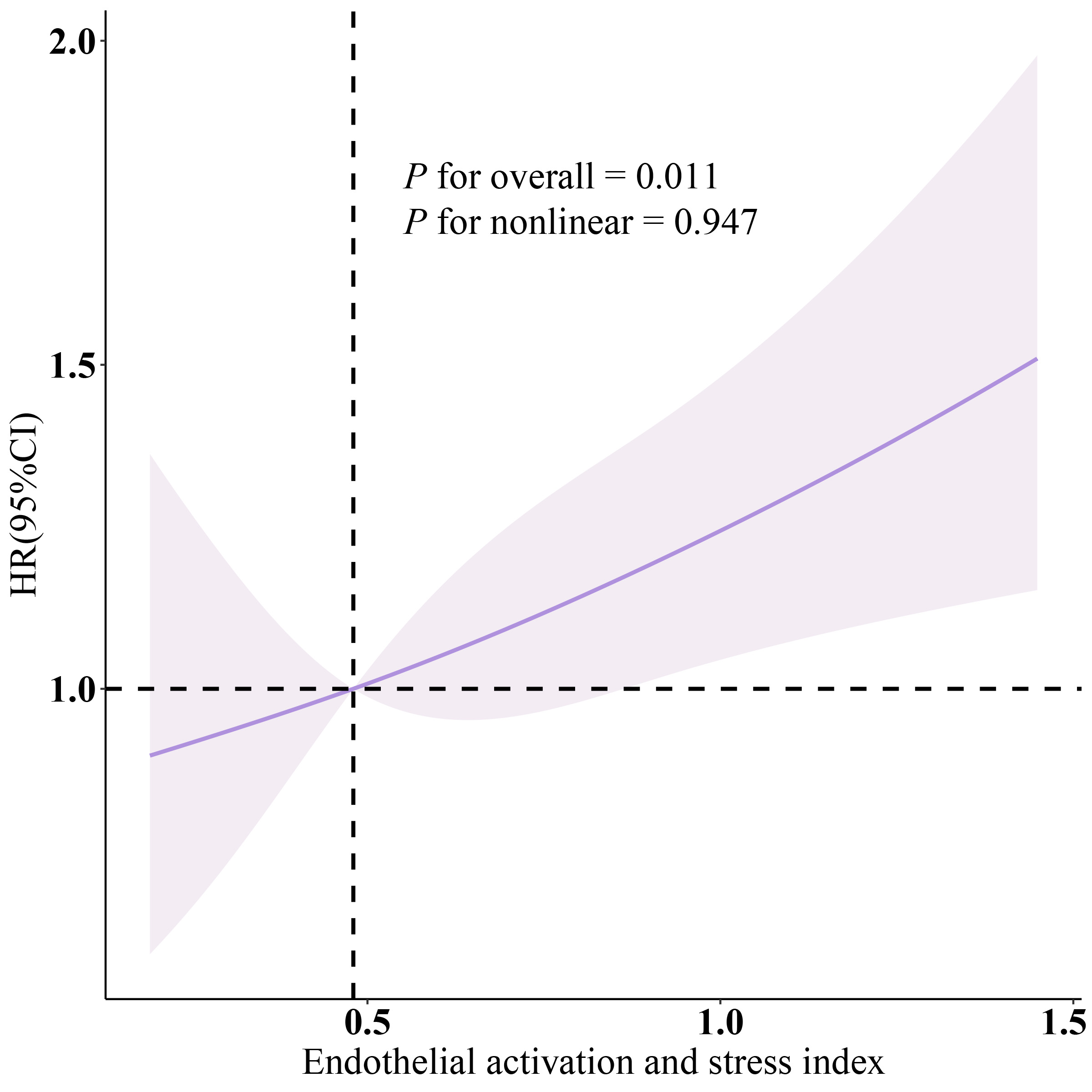


**Figure S1.** Dose-response relationships between EASIX and all-cause mortality in the BC population. OR (solid lines) and 95% confidence levels (shaded areas) were adjusted for age, education level, marital status, PIR, race, hypertension, diabetes, and hyperlipidemia.


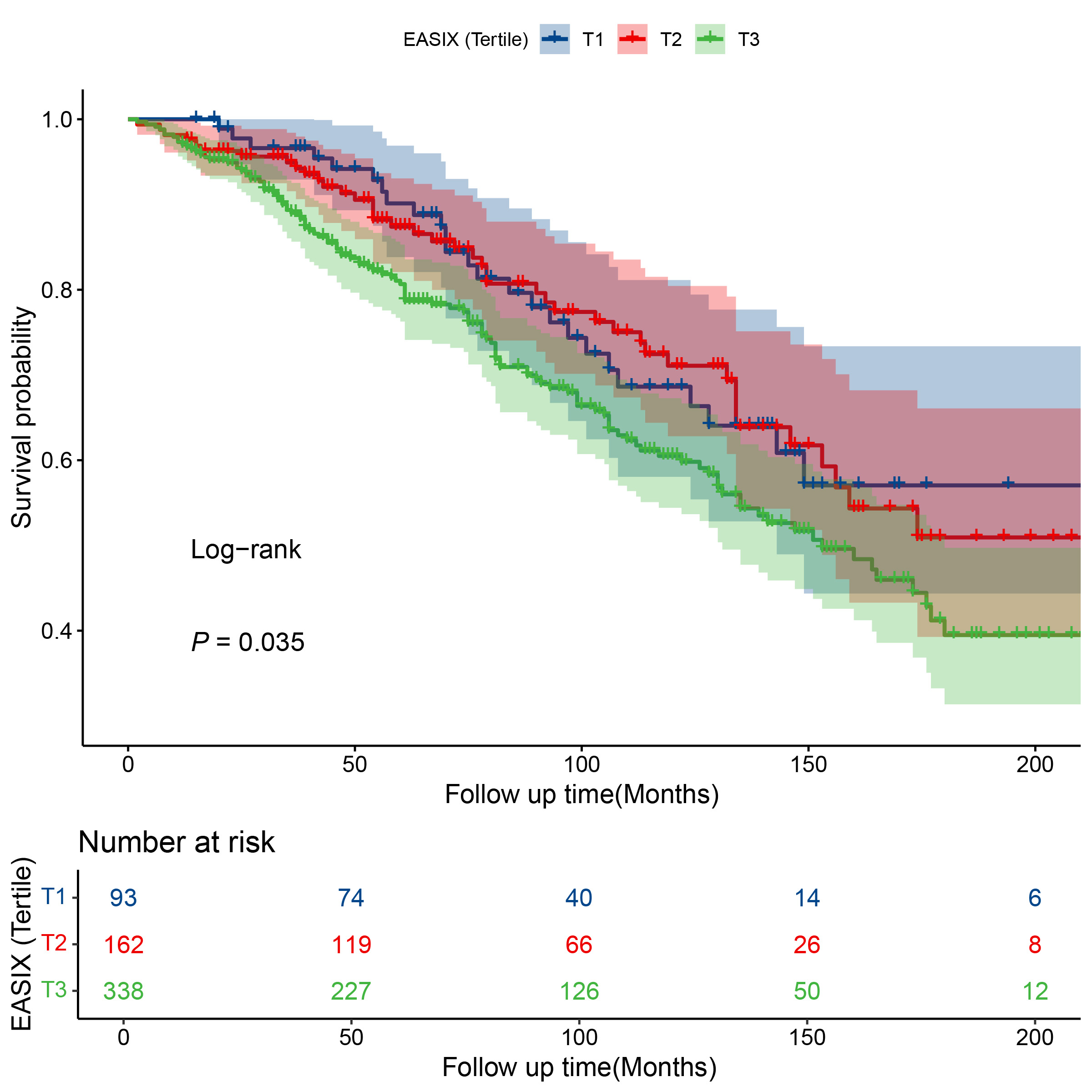


**Figure S2.** Kaplan–Meier analyses for all-cause mortality in the BC population among the EASIX groups.
